# Identification and Validation of Housekeeping Genes for Gene Expression Analysis of Cancer Stem Cells

**DOI:** 10.1371/journal.pone.0149481

**Published:** 2016-02-19

**Authors:** Silvia Lemma, Sofia Avnet, Manuela Salerno, Tokuhiro Chano, Nicola Baldini

**Affiliations:** 1 Laboratory for Orthopaedic Pathophysiology and Regenerative Medicine, Istituto Ortopedico Rizzoli, Bologna, Italy; 2 Department of Biomedical and Neuromotor Sciences, University of Bologna, Bologna, Italy; 3 Department of Clinical Laboratory Medicine, Shiga University of Medical Science, Otsu, Shiga, Japan; University of Navarra, SPAIN

## Abstract

The characterization of cancer stem cell (CSC) subpopulation, through the comparison of the gene expression signature in respect to the native cancer cells, is particularly important for the identification of novel and more effective anticancer strategies. However, CSC have peculiar characteristics in terms of adhesion, growth, and metabolism that possibly implies a different modulation of the expression of the most commonly used housekeeping genes (HKG), like b-actin (ACTB). Although it is crucial to identify which are the most stable HKG genes to normalize the data derived from quantitative Real-Time PCR analysis to obtain robust and consistent results, an exhaustive validation of reference genes in CSC is still missing. Here, we isolated CSC spheres from different musculoskeletal sarcomas and carcinomas as a model to investigate on the stability of the mRNA expression of 15 commonly used HKG, in respect to the native cells. The selected genes were analysed for the variation coefficient and compared using the popular algorithms NormFinder and geNorm to evaluate stability ranking. As a result, we found that: 1) Tata Binding Protein (TBP), Tyrosine 3-monooxygenase/tryptophan 5-monooxygenase activation protein zeta polypeptide (YWHAZ), Peptidylprolyl isomerase A (PPIA), and Hydroxymethylbilane synthase (HMBS) are the most stable HKG for the comparison between CSC and native cells; 2) at least four reference genes should be considered for robust results; 3) the use of ACTB should not be recommended, 4) specific HKG should be considered for studies that are focused only on a specific tumor type, like sarcoma or carcinoma. Our results should be taken in consideration for all the studies of gene expression analysis of CSC, and will substantially contribute for future investigations aimed to identify novel anticancer therapy based on CSC targeting.

## Introduction

Different populations of cells form malignant tumors. Within any given normal tissues, reside a subpopulation of stem cells with abilities of self-renewal and differentiation into specialized cells. Similarly, a tumor is composed of a heterogeneous population of malignant cells with distinct rank of differentiation, proliferation and tumorigenic potential. Among such heterogeneous malignant cells, cancer stem cells (CSC) are referred as a small but distinct population of element in the tumor mass that are primary involved in the steps of initiation, transformation and subsequent progression of the tumor [1]. The CSC model argues that, like stem cells of normal tissues, tumor cells follow hierarchical organizations in which CSC lying at the apex hold the capacity for tumorigenesis [2], metastasis promotion [3–5], and resistance to chemotherapy or radiotherapy [6–8]. This tumor-initiating cell population was isolated and characterized for the first time in human myeloid leukaemia [9,10], and subsequently also in other solid tumors [11–13]. The most widely used assay for the isolation of CSC is the sphere-forming assay and is based on the ability of CSC to grow in anchorage-independent conditions and to form floating colonies [12], the so-called “spheres”. Previously, we isolated CSC spheres from human musculoskeletal sarcomas [14–16], and in this study we also isolated CSC from different carcinoma. Whereas carcinomas are common adult malignancies that display high metastatic index at diagnosis and extensive morbidity [7, 12], musculoskeletal sarcomas are heterogeneous, relatively rare, and highly aggressive malignancies of bone and soft tissues that frequently occur in children and young adults [17]. The high rate of relapse typical of these neoplasms dramatically affects the clinical outcome and, despite surgery can be curative, tumor prognosis remains poor. Therefore, current therapeutic approaches are not sufficient to improve the clinical outcome, and further improvements may derive only from a better understanding of molecular mechanisms of these diseases, and from the identification of specific markers that definitely distinguish CSC from other tumor cells. Thus, under this context, the *in vitro* isolation of spheres provides an invaluable tool.

Quantitative Real-Time Polymerase Chain Reaction (qRT-PCR) is the most sensitive and accurate method to quantify mRNA expression of a single gene in various experimental conditions, and requires normalization of data against a reference gene, which typically should have a highly stable expression under the different considered experimental procedures [18]. The identification of specific housekeeping genes (HKG) is a pivotal prerequisite for studying the relative change in mRNA expression of a target gene. The selected HKG should not be co-regulated with the target gene or influenced by the experimental procedure. It should also be expressed in abundance and have minimal variability. The most common method for normalizing gene expression levels is to compare the mRNA levels of the gene of interest to the endogenous control gene. Normalization of qRT-PCR data against random HKG may result in erroneous calculation of the normalization factor used to compare the experimental conditions, and therefore hiding biological differences among samples [19]. Among different reference genes, beta-Actin, Glyceraldehyde 3-phosphate dehydrogenase, or beta-Tubulin are the most commonly used, as they are highly expressed, necessary for survival, not-regulated by signalling pathways, and are synthesized in all nucleated cell types. However, recent findings demonstrated that even beta-Actin, one of the most commonly used HKG, could be an unsuitable internal control [20,21].

qRT-PCR analyses of CSC have already been performed but, for our knowledge, no justification to the selection of the HKG is still available. In this study, we selected 15 of the most used HKG to evaluate their stability in both CSC and adherent native cells isolated from human rhabdomyosarcoma (RS), osteosarcoma (OS), Ewing’s sarcoma (ES), breast carcinoma (BC) and renal carcinoma (RC). Through the comparison of the coefficient variation and the usage of geNorm [22] and NormFinder [23] softwares we performed a valid, reproducible, and comparative analysis of the stability of the selected HKG.

## Materials and Methods

### Native tumor cell lines and CSC cultures

RS cell line (RD), OS cell lines (MG-63, HOS, Saos-2), ES cell line (A-673), BC cell line (MDA-MB-231) and RC cell line (ACHN), were purchased from American Type Culture Collection (ATCC,Manassas, VA, USA), and cultured in Iscove’s modified Dulbecco’s medium (IMDM, Gibco), plus 20 U/mL penicillin, 100 mg/mL streptomycin, and 10% heat-inactivated fetal bovine serum (FBS) (complete IMDM) at 37°C in a humidified 5% CO_2_ atmosphere. A human primary ES culture (ES4540) was also used, and was obtained from a fresh biopsy of human ES, and characterized, as previously described [16]. The ES4540 sample was collected after a signed informed consent and following the approval of the Istituto Ortopedico Rizzoli ethics committee (0033626, 9 Nov 2011), according with the Declaration of Helsinki. Briefly, tissue samples were subjected to mechanical mincing, followed by enzymatic digestion, to obtain single cells that were seeded in complete IMDM until the formation of a monolayer.

CSC cells were obtained as previously described [16]. Briefly, all native tumor cell cultures were maintained in anchorage-independent conditions in DMEM:F12 medium with progesteron (20 nM), putrescin (10 mg/mL), sodium selenite (30 nM), apo-transferrin (100 μg/mL), and insulin (25 μg/mL) (Sigma-Aldrich, St. Louis, MO) in low-attachment flasks (Nunc, Penfield, NY) (sphere-forming assay). We obtained the CSC culture by maintaining the spheroid in anchorage-independent conditions in specific cell media, adding the growth factors EGF and bFGF every 3–4 days (twice at week). Fresh human EGF (20 ng/mL) and bFGF (10 ng/mL) (PeproTech, Rocky Hill, NJ) were added twice a week until cells started to grow as floating aggregates (spheres). Spheroid cultures were amplified by treating the primary CSC culture with trypsin, followed by gentle mechanical dissociation, and by re-plating single-cell suspension to obtain the second spheroid culture. Viability was verified by erythrosine staining. The percentage of dead cells was low (10–15% of dead cells). Only those cultures that were able to form spheres and that expressed stem cell-related markers were considered.

### Illumina genome analyzer sequencing and data analysis

A deep sequencing analysis of MG-63, HOS, and Saos-2 OS cell models was performed to compare the global transcriptional expression of CSC to the respective native cells, in order to select a panel of stable HKG for qRT-PCR analysis. Briefly, total RNA was collected from the cell lysate in acid guanidinium thiocyanate-phenol-chloroform [24]. The total RNA was quantified by Bioanalyzer (Agilent, Santa Clara, CA) following the manufacturer's instructions. RIN (RNA Integrity Number) and A260/A280 ratio of the prepared total RNA were all 10, and over 1.8, respectively. The library of template molecules for high throughput DNA sequencing was converted from the total RNA using TruSeq RNA Sample Prep Kit v2 (Illumina, San Diego, CA), following the manufacturer's protocol. The library was also quantified with Bioanalyzer (Agilent), following the manufacturer's instruction. The library (7 pM) was subjected to cluster amplification on a Single Read Flow Cell v4 with a cluster generation instrument (Illumina). Sequencing was performed on a Genome Analyzer GAIIx for 70 cycles using Cycle Sequencing v4 regents (Illumina). Human genome build 19 (hg19) were downloaded from University of California, Santa Cruz genome browser (http://genome.ucsc.edu/). Image analysis and base calling were performed using Off-Line Basecaller Software 1.6 (Illumina). Reads were aligned using ELAND v2 of CASAVA Software 1.7 with the sequence data sets. Transcript coverage for every gene locus was calculated from the total number passing filter reads that mapped, by ELAND-RNA, to exons. These analyses were performed using default parameters. The data were viewed using Genome Studio Software (Illumina). The advanced analysis for quantification with Quantile normalization algorithm was performed using Avadis NGS software (version1.5, Strand Scientific Intelligence Inc., San Francisco, CA).

### RNA isolation and cDNA synthesis

Total RNA was extracted from CSC and native cells from all the different histotypes included in the study by using the NucleoSpin RNA II (Macherey-Nagel, Düren, Germany). On-column DNase digestion was performed following manufacturer’s instructions. The total RNA purity was quantified using a Nanodrop Spectrophotometer (NanoDrop Technologies). Total RNA (0.5 μg) were reverse-transcribed into cDNA in 20 μl final volume, using MuLV Reverse Transcriptase and RNase inhibitor (Applied Biosystems, Foster City, Ca, USA). First-strand cDNA was synthesized using random hexamers. For each sample, 3 biological replicates were processed.

### qRT-PCR

qRT-PCR was performed by using a Light Cycler instrument and the Universal Probe Library system (Roche Applied Science, Monza, Italy). Probe and primers were selected by using a web-based assay design software (ProbeFinder https://www.roche-applied-science.com), and were further controlled using Oligo Primer Analysis Software. Only primers spanning an exon-exon junction and producing a PCR amplificate with length between 70 and 150 base pairs were selected. All the primers designed were analysed by BLAST to verify their specificity (National Center for Biotechnology Information). All cDNA were diluted 1:10, and 10 μl were used as template and included in a 20 μl of total volume of qRT-PCR reaction. The protocol of amplification was: 95°C for 10 min; 95°C for 10 s, 60°C for 30 s, and 72°C for 1 s for 45 cycles; 40°C for 30 s. Each assay included a blank. Table 1 provides a summary of all the HKG, primer sequences, and probes included in this study. For the evaluation of the expression of c-Myc (NM_002467.4), KLF4 (NM_004235.4), Nanog (NM_0248695.2) and OCT3/4 (NM_002701.4), the following primers were used: c-Myc-F 5’-gctgcttagacgctggattt-3’, c-Myc-R 5’-taacgttgaggggcatcg-3’, probe 66; KLF4-F 5’-ccatctttctccacgttcg-3’, KLF4-R 5’-agtcgcttcatgtgggagag-3’, probe 7;. Nanog-F 5’-ATGCCTCACACGGAGACTGT-3’, Nanog-R 5’-AGGGCTGTCCTGAATAAGCA-3, probe 69; OCT3/4-F 5’-CTTCGCAAGCCCTCATTTC-3’, OCT3/4-R 5’-GAGAAGGCGAAATCCGAAG-3’, probe 60. For the purpose of normalization, the relative expression of KLF4, c-Myc, Nanog and OCT3/4 were normalised by the reference gene ACTB or for the geometric average of YWHAZ and GAPDH for CSC and native cells from MG-63, or for the geometric average of PPIA and HMBS for CSC and native cells from ACHN and MDA-MB-231. The relative expression of the stem cell markers was calculated using the ΔΔCt model [25].

**Table 1:** Candidate HKG genes.

Gene	Full name	Function	Accession Number	Primers	Probe
**18S rRNA**	18S ribosomal RNA	Structural RNA for the small component of eukaryotic cytoplasmic ribosomes.	X03205.1	F = gcaattattccccatgaacg R = gggacttaatcaacgcaagc	48
**ACTB**	Actin, beta	Cytoskeletal structural protein	NM_001101.2	F = ccaccgcgagaagatga R = ccagaggcgtacagggatag	64
**B2M**	Beta-2-microglobulin	Component of the class I major histocompatibility complex (MHC)	NM_004048.2	F = ttctggcctggaggctatc R = tcaggaaatttgactttccattc	42
**G6PD**	Glucose-6-phosphate dehydrogenase	Produces pentose sugars for nucleic acid synthesis and main producer of NADPH reducing power	M24470.1|M24470	F = gaagggccacatcatctctg R = atctgctccagttccaaagg	75
**GAPDH**	Glyceraldehyde 3-phosphate dehydrogenase	Oxidoreductase in glycolysis and gluconeogenesis	NM_002046.3	F = agccacatcgctcagacac R = gcccaatacgaccaaatcc	60
**GUSB**	Beta-glucuronidase	Hyrolase that degrades glycosaminoglycans	M15182.1|M15182	F = cgccctgcctatctgtattc R = tccccacagggagtgtgtag	57
**HMBS**	Hydroxymethylbilane synthase	Third enzyme in the heme biosynthetic pathway	NM_000190.3	F = tgtggtgggaaccagctc R = tgttgaggtttccccgaat	26
**HPRT1**	Hypoxanthine phosphoribosyltransferase 1	Purine synthesis in salvage pathway	M31642.1|M31642	F = tgaccttgatttattttgcatacc R = cgagcaagacgttcagtcct	73
**PGK1**	Phosphoglycerate kinase 1	Transferase in glycolysis and gluconeogenesis	NM_000291.3	F = ggagaacctccgctttcat R = gctggctcggctttaacc	69
**PPIA**	Peptidylprolyl isomerase A (cyclophilin A)	Isomerase involved in the cis-trans isomerization of peptide bonds in oligopeptides	NM_021130.3	F = atgctggacccaacacaaat R = tctttcactttgccaaacacc	48
**RPL13a**	Ribosomal protein L13a	Structural component of the large 60S ribosomal subunit	NM_012423.3	F = caagcggatgaacaccaac R = tgtggggcagcatacctc	28
**SDHA**	Succinate dehydrogenase complex, subunit A, flavoprotein (Fp)	Electron transporter in the TCA cycle and respiratory chain	NM_004168.2	F = ggacctggttgtctttggtc R = ccagcgtttggtttaattgg	80
**TBP**	TATA-binding protein	General RNA polymerase II transcription factor	NM_001172085.1	F = ttgggttttccagctaagttct R = ccaggaaataactctggctca	24
**TUBB**	Tubulin, beta class I	Major constituent of microtubules	NM_178014.2	F = ataccttgaggcgagcaaaa R = tcactgatcacctcccagaac	64
**YWHAZ**	Tyrosine 3-monooxygenase/tryptophan 5-monooxygenase activation protein zeta polypeptide	Belongs to the 14-3-3 family of protein which mediate signal transduction	NM_003406.3	F = ccgttacttggctgaggttg R = tgcttgttgtgactgatcgac	9

### NormFinder analysis

NormFinder program is a VBA applet [23] based on a variance estimation approach, which ranks the candidate HKG based on their stability evaluation, and assigns a stability value to each candidate gene using a model-based approach. In agreement with NormFinder requirements, the Ct values were transformed in relative quantity, using the lowest Ct value as calibrator. According to the analysis, the lowest stability value was top ranked. We grouped all the data in 3 different clusters: 1) all data from CSC of different tumors; 2) all data from native cells of different tumors; 3) all data from different tumors with CSC and native cells pooled together. For the third group of data, in addition to CSC and native cells obtained from all tumors pooled together, we also considered CSC and native cells obtained from the single tumor type, from sarcoma or from carcinoma. All results were obtained from 3 sets of replicates.

### GeNorm analysis

GeNorm v. 3.0 [22] available in qbase+ [26] (Biogazelle, Ghent University, Belgium, http://www.qbaseplus.com) was used to evaluate the stability of candidate HKG. GeNorm calculates all the possible average pairwise variation between the candidate genes and provides a measure of the expression stability (M) of each gene. An M-value below 1.5 indicates stable HKG. The candidate reference gene with the lowest M value was considered to have the most stable expression. GeNorm ranks candidate reference genes on the basis of their stability of expression, and performing stepwise exclusion of the gene with the highest M-value (the least stable expressed gene), and recalculates M-values for the remaining genes. We use also GeNorm to verify if a single HKG is sufficient for an adequate normalization. Indeed, GeNorm provides the optimal number of reference genes required for accurate normalization. V values below the cut-off value 0.15 indicated the optimal number of genes required for data normalization. Similarly to the analysis performed with NormFinder, we grouped all the data and the results in 3 different clusters. All results were obtained from 3 sets of replicates.

### Coefficient of variation analysis

Gene expression stability evaluated by the coefficient of variation (CV) was calculated by dividing the standard deviation (SD) of threshold cycles (Ct) by the mean Ct value. As in the analysis performed with NormFinder and GeNorm, we grouped all the data in 3 different clusters, and the results were obtained from 3 sets of replicates.

### Rank aggregation

The analyses performed by the three described methods showed some differences in the stability rank of the HKG. Therefore, we identified the most stable genes by considering the lowest value of the mathematic average of the NormFinder, geNorm and CV method ranks for every single gene.

### Statistical analysis

Statistical analysis was performed with StatView™ 5.0.1 software (SAS Institute Inc., Cary, NC). For the characterization of CSC and native cells from MG-63, MDA-MB-231 and ACHN, data were considered as not normally distributed, and nonparametric Mann-Whitney U test were used. Results were reported as mean ± standard error of mean (SEM). Standard deviation (SD) of delta Cycle threshold (ΔCt) values was calculated as pooled standard deviation (SDpooled). HKG expression variation in CSC and native cells was evaluated with the paired Wilcoxon signed-rank test. For all the analyses, differences were considered significant with a *p-*values ≤ 0.05.

## Results

### RNA quality control and characterization of CSC

We established sphere cultures from commercially available cell lines from 3 different sarcoma and 2 carcinoma histotypes (MG-63 for OS, RD for RS, A-673 for ES, MDA-MB-23 for BC, and ACHN for RC), and from a fresh ES biopsy (ES4540). The spectrophotometric analysis confirmed the purity of the samples, with an A_260_/_280_ ratio of 2.08 ± 0.06, indicating protein-free pure RNA, and a A_260/230_ ratio of 1.98 ± 0.21, indicating that the total RNA was phenol and ethanol free.

**Fig 1:**
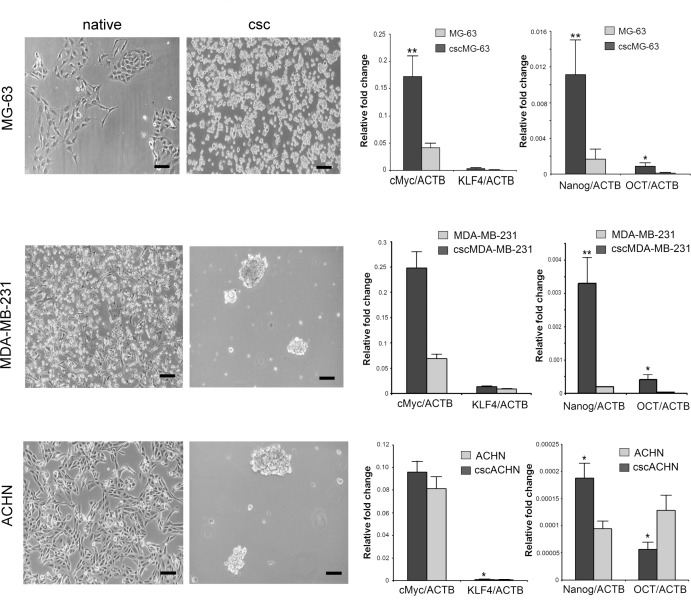
Characterization of sarcospheres obtained from MG-63. Representative images of adherent native cells and CSC floating spheres observed by inverted optical microscope for the different cell lines (scale bar 100 μm, left panel), and gene expression of stem cell markers by qRT-PCR (right panel). Normalization to ACTB. For cscMG-63, c-Myc **p = 0.0019, KLF4 p = ns, Nanog **p = 0.0010, OCT3/4 *p = 0.0265. For cscMDA-MB-231, c-Myc **p = 0.0011, KLF4 *p = 0.0130, Nanog **p = 0.0011, OCT3/4 *p = 0.0325. For cscACHN, c-Myc p = ns, KLF4 *p = 0.0130, Nanog *p = 0.0130. For adherent ACHN, OCT3/4 *p = 0.0130. (n = 6–12).

The stemness-like features for all the CSC cultures included in this study were previously characterized [16,27], with the exception of cscMG-63, cscMDA-MB-231, and cscACHN for which the ability to growth as floating aggregates (Fig 1), and the mRNA expression for KLF4, c-Myc, Nanog, and OCT3/4 stemness markers [28] were here confirmed.

In particular, in cscMG-63 we found a trend of increased expression of KLF4 (Fig 1; n = 12; p = ns), and a significantly higher expression of c-Myc (**p = 0.0019), Nanog (**p = 0.0010), and OCT3/4 (*p = 0.0265). CSC from MDA-MB-231 highly express all the stamness markers than the adherent culture (Fig 1; n = 6; KLF4 *p = 0.0130; c-Myc **p = 0.0011; Nanog **p = 0.0011; OCT3/4 *p = 0.0325), whereas the sphere cultures from ACHN expressed consistent levels of mRNA for KLF4 and Nanog (Fig 1; n = 6; KLF4 *p = 0.0130; Nanog *p = 0.0130), and no different expression for c-Myc. In cscACHN, the expression of the OCT3/4 marker is lower than the adherent native culture (Fig 1; *p = 0.0130). The stemness genes were normalised by ACTB, one of the most commonly used HKG.

### Expression profile of the candidate HKG genes

Fifteen candidate reference genes (Table 1) were selected through the analysis of literature survey on studies on normal stem cells and tumor cells with qRT-PCR analysis.

**Fig 2:**
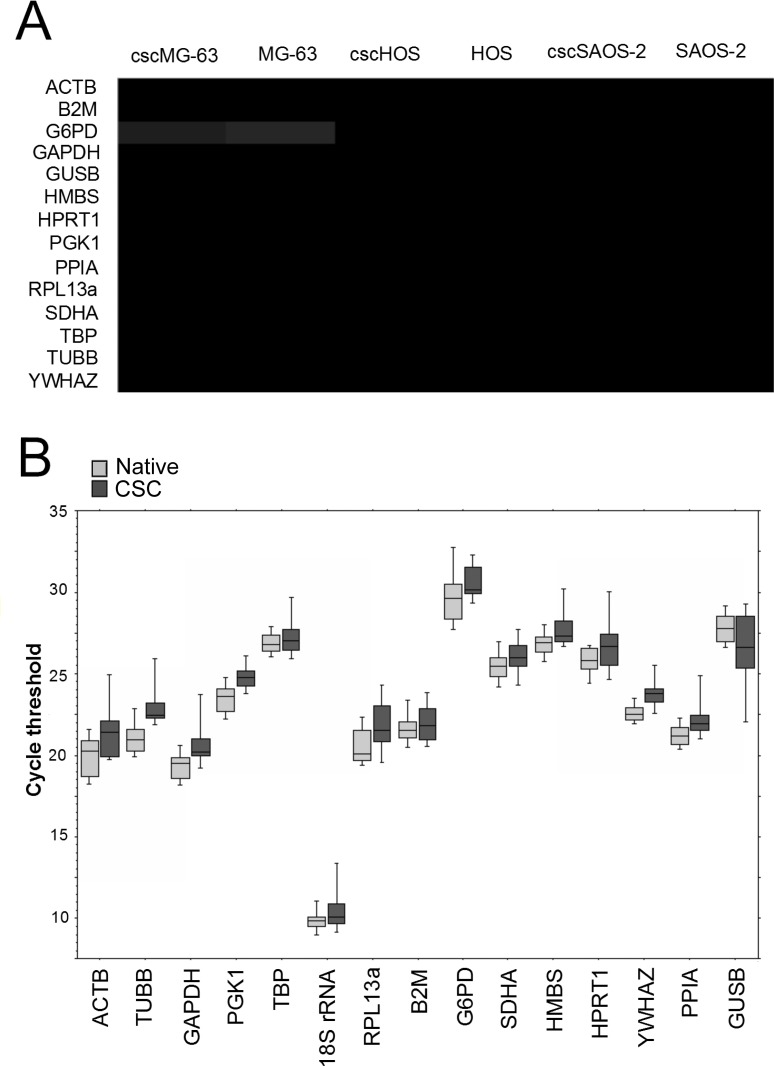
Transcription profiling of the selected reference genes. (A) Heat map showing the relative expression of the selected genes in native cells and CSC from MG-63, HOS, and SAOS-2. (B). Transcriptional profile of Cycle threshold (Ct) values of candidate HKG genes in CSC and native cell lines. Boxes represent lower and upper quartiles of cycle threshold range with the median indicated, vertical bars represent the 10^th^ and 90^th^ percentiles. In both CSC and adherent cell lines, 18S rRNA was the most highly expressed gene (lower Ct value), whereas G6PD was the least expressed gene (highest Ct value).

We selected those genes that belong to different functional classes or pathways in order to reduce the probability to include in the analysis co-regulated genes. The transcript profiling of the selected genes was preliminary analysed by Illumina Genome Analyzer sequencing. The deep sequencing analysis showed that the putative reference genes were stably expressed, with the exception of G6PD (Table 2, Fig 2A).

**Table 2:** Transcriptome data from deep sequencing analysis of CSC and native cells of osteosarcoma.

Gene	Ensembl ID	Entrez ID	cscMG-63	MG-63	cscHOS	HOS	cscSAOS-2	SAOS-2
**ACTB**	ENSG00000075624	60	13.565	13.881	13.906	14.208	13.946	14.120
**B2M**	ENSG00000166710	567	13.117	13.172	12.706	12.564	12.788	13.071
**G6PD**	ENSG00000160211	2539	10.290	8.851	11.059	10.190	11.559	10.925
**GAPDH**	ENSG00000111640	2597	13.385	13.511	13.159	13.001	13.385	12.827
**GUSB**	ENSG00000169919	2990	9.778	9.271	10.630	9.549	11.996	10.151
**HMBS**	ENSG00000149397	3145	10.035	9.041	10.511	10.912	9.905	9.744
**HPRT1**	ENSG00000165704	3251	9.304	9.509	10.125	10.475	10.190	10.093
**PGK1**	ENSG00000102144	5230	12.137	12.440	12.159	12.320	12.895	12.663
**PPIA**	ENSG00000196262	5478	10.804	11.126	11.472	11.950	10.268	10.620
**RPL13a**	ENSG00000142541	23521	11.081	11.589	11.315	11.732	11.273	11.297
**SDHA**	ENSG00000073578	6389	11.096	10.548	11.476	11.326	11.813	11.232
**TBP**	ENSG00000112592	6908	9.238	8.985	8.922	9.017	9.285	9.113
**TUBB**	ENSG00000196230	203068	13.866	14.479	14.251	14.378	14.059	14.590
**YWHAZ**	ENSG00000164924	7534	12.379	13.023	11.812	12.737	12.437	12.641

We used only MG-63 as representative of the OS histotype for the following qRT-PCR analysis to confirm the data obtained by deep sequencing. To compare different mRNA transcription levels we used the threshold cycles (Ct) values. Ct value is the intersection between an amplification curve and a threshold line, and is inversely correlated with the amount of target gene present in the PCR reaction [29]. The 15 candidate reference genes exhibited a broad range of expression level. The distribution of median Ct values and percentile for each gene is shown in the blox plot of Fig 2B. Reference genes were less expressed in CSC compared to native cells. The smallest differences in gene expression between CSC and native cell lines, expressed as ΔCt, were detected for B2M, TBP and SDHA, whereas the highest ΔCt were calculated for ACTB, TUBB and PGK1 (Table 3). By performing the paired Wilcoxon signed-rank test for each gene to evaluate the difference between CSC and native cells obtained from the same cells of origin, we found a significant difference in HKG expression between CSC and native cells for around half of the HKG examined (Table 3).

**Table 3:** Ct values of candidate HKG genes in CSC and native cell lines.

Gene	Ct values CSC [mean ± SD]	Ct values native cell lines [mean ± SD]	ΔCt values CSC and native cells [mean ± SDpooled]	P value
**18S rRNA**	10.51 ± 1.53	9.89 ± 0.84	0.62 ± 0.24	n.s.
**ACTB**	21.58 ± 1.98	19.96 ± 1.42	1.62 ± 0.64	0.0277
**B2M**	21.95 ± 1.22	21.63 ± 1.13	0.32 ± 0.20	n.s.
**G6PD**	30.61 ± 1.14	29.82 ± 2.13	0.79 ± 0.61	n.s.
**GAPDH**	20.72 ± 1.54	19.43 ± 1.01	1.29 ± 0.46	0.0277
**GUSB**	28.66 ± 2.16	27.81 ± 1.04	0.85 ± 0.34	n.s.
**HMBS**	27.82 ± 1.30	26.85 ± 0.87	0.97 ± 0.34	n.s.
**HPRT1**	26.82 ± 1.88	25.84 ± 1.01	0.98 ± 0.37	0.0277
**PGK1**	24.80 ± 0.81	23.43 ± 0.98	1.37 ± 0.48	0.0277
**PPIA**	22.27 ± 1.36	21.24 ± 0.77	1.03 ± 0.35	0.0464
**RPL13a**	21.79 ± 1.64	20.51 ± 1.19	1.28 ± 0.49	0.0464
**SDHA**	26.01 ± 1.17	25.58 ± 1.09	0.44 ± 0.22	n.s.
**TBP**	27.32 ± 1.28	26.94 ± 0.89	0.37 ± 0.17	n.s.
**TUBB**	23.00 ± 1.66	21.07 ± 1.07	1.93 ± 0.65	0.0277
**YWHAZ**	23.83 ± 1.07	22.60 ± 0.77	1.23 ± 0.40	0.0277

n.s. not significant.

### Determination of housekeeping gene expression stability

HKG expression stability was evaluated by using NormFinder VBA applet, the GeNorm software, and by calculating and comparing the coefficient of variation (CV) in three different groups: in (1) CSC or (2) native cells, with the aim to identify the most stable genes within a specific cell subtypes, and (3) in the pooled CSC and native cells, with the aim to identify the most stable genes to be considered as reference genes for the comparison of gene expression between CSC and native cells.

The stability values for NormFinder and M values for GeNorm are parameters of stability that are inversely correlated to the expression stability of the HKG. All the 15 candidate reference genes had a M value lower than the threshold value of 1.5 (Table 4), which indicated that all can be considered as acceptable in terms of stability [22].

**Table 4:** Ranking of the stability of the expression of candidate reference genes by NormFinder, geNorm, and CV analyses.

Group of cells	Gene	NormFinder		GeNorm		Coefficient of Variation (CV)	
		Stability value	Rank	M value	Rank	CV	Rank
**CSC**	GAPDH	0.059	1	0.500	2	0.074	11
	PGK1	0.077	2	0.706	8	0.033	1
	HMBS	0.095	3	0.627	7	0.047	5
	YWHAZ	0.101	4	0.516	3	0.045	3
	PPIA	0.102	5	0.528	4	0.061	8
	G6PD	0.104	6	0.761	9	0.037	2
	TUBB	0.119	7	0.611	6	0.072	10
	ACTB	0.138	8	1.095	14	0.092	14
	HPRT1	0.149	9	0.921	12	0.070	9
	SDHA	0.151	10	0.799	10	0.045	4
	B2M	0.153	11	1.179	15	0.055	7
	TBP	0.160	12	0.456	1	0.047	6
	RPL13a	0.162	13	0.850	11	0.075	13
	18S rRNA	0.178	14	0.583	5	0.146	15
	GUSB	0.282	15	1.022	13	0.075	12
**Native cells**	18S rRNA	0.154	1	0.290	1	0.085	15
	TBP	0.158	2	0.498	4	0.033	2
	PPIA	0.159	3	0.296	2	0.036	4
	SDHA	0.168	4	0.653	7	0.043	8
	GAPDH	0.183	5	0.345	3	0.052	10
	B2M	0.187	6	0.689	8	0.052	11
	HMBS	0.190	7	0.786	11	0.032	1
	HPRT1	0.191	8	0.729	9	0.039	6
	YWHAZ	0.199	9	0.554	5	0.034	3
	TUBB	0.201	10	0.596	6	0.051	9
	GUSB	0.207	11	0.762	10	0.037	5
	PGK1	0.219	12	0.809	12	0.042	7
	RPL13a	0.226	13	0.961	14	0.058	12
	G6PD	0.229	14	1.092	15	0.071	14
	ACTB	0.257	15	0.888	13	0.071	13
**CSC and native cells (pooled)**	GAPDH	0.124	1	0.484	3	0.086	12
	PPIA	0.134	2	0.437	1	0.066	8
	HMBS	0.141	3	0.689	6	0.051	4
	TBP	0.149	4	0.624	5	0.046	1
	PGK1	0.153	5	0.786	8	0.052	5
	YWHAZ	0.161	6	0.588	4	0.055	6
	G6PD	0.165	7	1.167	15	0.067	10
	18S rRNA	0.168	8	0.470	2	0.143	15
	TUBB	0.168	9	0.735	7	0.091	13
	SDHA	0.172	10	0.821	9	0.050	3
	HPRT1	0.173	11	0.858	10	0.066	9
	B2M	0.182	12	1.037	13	0.050	2
	ACTB	0.192	13	1.104	14	0.103	14
	RPL13a	0.198	14	0.979	12	0.074	11
	GUSB	0.238	15	0.918	11	0.066	7

**Fig 3:**
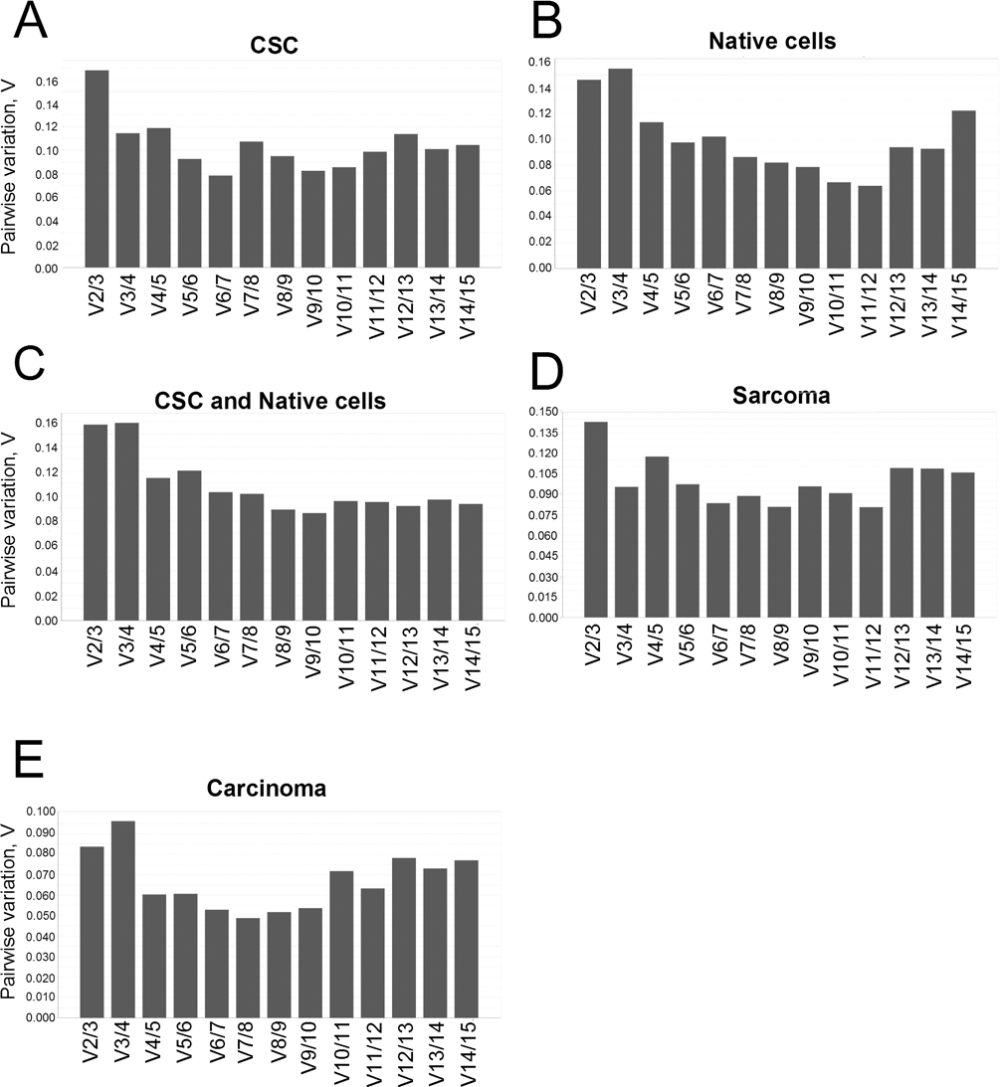
GeNorm evaluation of the minimal number of genes and validation of the identified top-ranked HKG genes for qRT-PCR normalization. The minimal number of genes required for data normalization was evaluated by pairwise variation (Vn/n +1) in (A) CSC, (B) native cells, (C) in the CSC and native cell lines from all tumors, (D) from sarcoma and (C) from carcinomas. A variation coefficient (V) below 0.15 indicates the optimal number of genes required for data normalization. V2/3<0.15 indicates that 2 genes are required for data normalization.

1) The most stable HKG in CSC. Using the NormFinder VBA Applet, the 3 most stable HKG resulted GAPDH, PGK1 and HMBS (from the first to the third stable), whereas the less stable were RPL13a, B2M and GUSB. Using GeNorm software, we identified YWHAZ, GAPDH and TBP as the most stable HKG, whereas the less stable genes were ACTB, GUSB and B2M. CV method also underlined that the use of ACTB should be avoided, whereas, like for GeNorm and NormFinder analyses, the use of PGK1 and YWHAZ is recommended. A comparison of the ranking produced by the three approaches revealed difference depending on the type of algorithm applied. The minimal number of reference genes required for normalization was determined by GeNorm calculation of pairwise variation (variation coefficient, V) between a given number of genes and the inclusion of an additional gene, and the optimal number of reference gene was calculated as 3 (V3/4 0.114, Fig 3A). In conclusion, for the gene expression analyses of mRNA isolated from CSC, the optimal normalization factor should be calculated as the geometric mean of the reference targets YWHAZ, GAPDH and PGK1.

2) The most stable HKG in native adherent cells. NormFinder analysis revealed that 18S rRNA, TBP, and PPIA were the three best HKG, whereas RPL13a, G6PD, and ACTB were worse in expression stability. Similarly, GeNorm identified PPIA and 18S rRNA as the two most stable HKG, followed by GAPDH, whereas the less stable genes, in order from the last ranked, were G6PD, RPL13a, and ACTB. The CV method suggested HMBS, TBP and YWHAZ as the three most stable genes. The GeNorm calculation of the variation coefficient V suggested that the optimal number of reference genes was 2 (V2/3 0.146; Fig 3B), and the addition of a third reference gene resulted in a small effect on normalization (below the cut-off value of 0.15). In conclusion, for the native cell, according to the analyses, the optimal normalization factor should be calculated as the geometric mean of PPIA and TBP.

3) Finally we performed the analysis of CSC and native pooled cells in all tumors (A), in sarcoma (B) and carcinoma (C).

(3A) In CSC and native cells from all tumors, NormFinder identified GAPDH, TBP, and PPIA as the three most stable HKG, whereas ACTB, RPL13a and GUSB were the less stable. PPIA and GAPDH were also confirmed by GeNorm analysis, along with 18S rRNA. Again, ACTB was the last gene in stability for the GeNorm analysis, along with B2M and G6PD. The CV evaluation suggested TBP, B2M, and SDHA (Table 4), whereas ACTB, TUBB and 18S rRNA were the less stable. GeNorm recommended the use of 4 HKG for accurate gene expression analysis (V < 0.15 when comparing a normalization factor based on the 4 or 5 most stable targets; Fig 3C). Consequently, the normalization factor should be calculated as the geometric mean of TBP, PPIA, HMBS, and YWHAZ or GAPDH.

(3B) In CSC and native cells from sarcoma, the NormFinder software identified GAPDH, YWHAZ and 18S rRNA as the most stable genes, instead G6PD, RPL13a and B2M were the less stable. GAPDH and YWHAZ were identified as the best HKG also by the GeNorm analysis, whereas ACTB and RPL13a were among the last ranked genes. The CV method showed that HMBS, TBP, and SDHA had the best rank position (Table 5). The optimal number of reference targets is 2 (V2/3 0.143, Fig 3D). In conclusion, the optimal normalization factor can be calculated as the geometric mean of the reference targets YWHAZ and GAPDH.

**Table 5:** Ranking of the stability of the expression of candidate reference genes by NormFinder, geNorm, and CV analyses in CSC and native cells from carcinoma and sarcoma tumors.

CSC and native cells (pooled)	Gene	NormFinder		GeNorm		Coefficient of Variation (CV)	
		Stability value	Rank	M value	Rank	CV	Rank
Sarcoma	GAPDH	0.299	1	0.386	1	0.049	9
	YWHAZ	0.346	2	0.434	3	0.036	4
	18S rRNA	0.349	3	0.573	6	0.078	15
	PPIA	0.368	4	0.446	4	0.036	5
	TUBB	0.478	5	0.420	2	0.049	10
	TBP	0.489	6	0.607	7	0.033	2
	SDHA	0.489	7	0.525	5	0.035	3
	PGK1	0.503	8	0.656	8	0.037	6
	HMBS	0.562	9	0.697	9	0.031	1
	HPRT1	0.722	10	0.770	10	0.045	8
	GUSB	0.754	11	0.880	12	0.043	7
	ACTB	0.771	12	1.160	15	0.077	14
	B2M	0.826	13	0.834	11	0.059	11
	RPL13a	0.894	14	0.978	13	0.073	13
	G6PD	0.907	15	1.074	14	0.059	12
Carcinoma	PPIA	0.055	1	0.150	1	0.069	9
	HMBS	0.072	2	0.453	10	0.060	5
	RPL13a	0.088	3	0.193	3	0.075	11
	ACTB	0.091	4	0.390	8	0.092	13
	HPRT1	0.093	5	0.368	7	0.073	10
	18S rRNA	0.093	6	0.164	2	0.144	15
	TBP	0.094	7	0.417	9	0.053	2
	GAPDH	0.103	8	0.298	4	0.088	12
	GUSB	0.104	9	0.315	5	0.066	7
	G6PD	0.140	10	0.521	11	0.054	3
	B2M	0.140	11	0.785	15	0.036	1
	YWHAZ	0.160	12	0.341	6	0.067	8
	TUBB	0.160	13	0.713	14	0.103	14
	PGK1	0.182	14	0.647	13	0.064	6
	SDHA	0.219	15	0.572	12	0.059	4

(3C) The analysis of HKG stability in carcinoma revealed that PPIA, HMBS and RPL13a were the most stable HKG for NormFinder. GeNorm also confirmed PPIA and RPL13a as most stable targets by GeNorm, followed by 18S rRNA, whereas the CV method suggested B2M, TBP and G6PD (Table 5). The GeNorm calculation of the coefficient V suggested that 2 HKG are sufficient for normalization (V2/3 0.084, Fig 3E). In conclusion, the optimal normalization factor in this case should be calculated as the geometric mean of two of the following genes, PPIA, HMBS or RPL13a, which have the same overall rank value.

### Validation of the identified HKG in the CSC model

In gene expression evaluation, the use of suboptimal HKG can generate erroneous results or can hide a difference in gene expression. This is particularly important for genes that slightly change between two populations of cells, as CSC and native cells.

**Fig 4:**
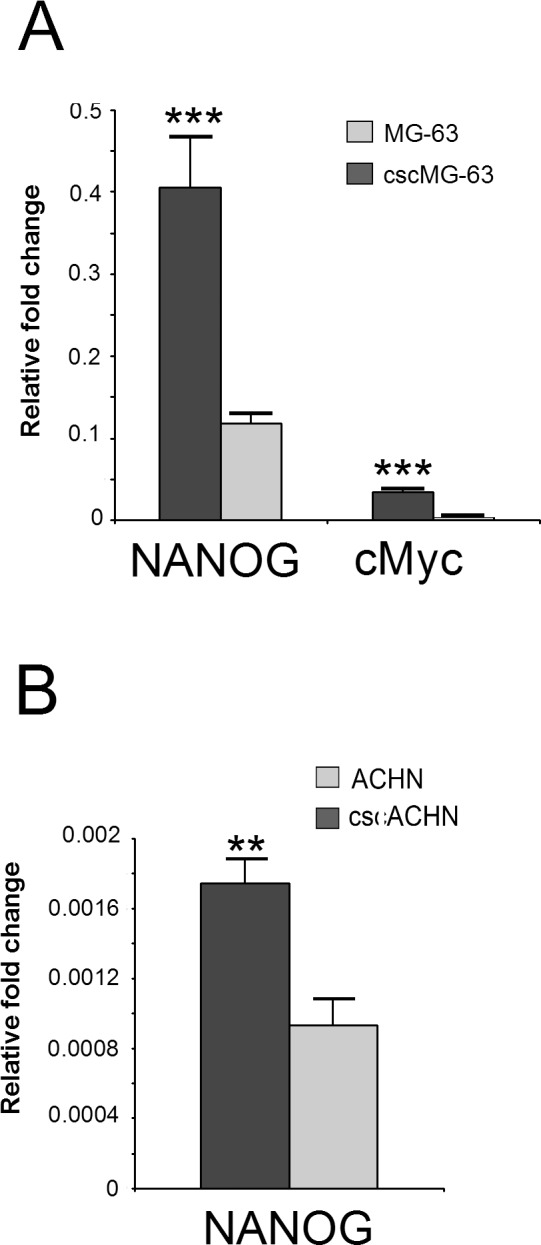
Validation of the identified housekeeping genes. The effect of gene expression normalization with optimal HKG was investigated on CSC and native cells from MG-63 and ACHN. (A) For the osteosarcoma cell line, the expression of the stem cell markers Nanog and cMyc were evaluated and normalized to the geometric mean of GAPDH and YWHAZ. ***p = 0.0007 for Nanog, ***p = 0.0003 for c-Myc. (B) For the ranal carcinoma cell line, the expression of Nanog and cMyc was normalized to the geometric mean of PPIA and HMBS. **p = 0.0043 for Nanog. (n = 6).

We analyzed the expression of the stemness genes c-Myc, KLF4, Nanog, and OCT3/4 that were previously normalized to ACTB (Fig 1), with the identified top-ranking HKG for CSC and native cells, in sarcoma and carcinoma, respectively (GAPDH and YWHAZ for sarcoma, and PPIA and HMBS for carcinoma). Some of the stem-related genes considered showed an improved robustness of statistical analysis performed with the normalization with the most stable HKG, in respect with ACTB. In particular, as shown in Fig 4, we found that the normalization of Nanog to the geometric mean of the most stable HKG resulted in ***p = 0.0007 for cscMG-63 and **p = 0.0043 for cscACHN, whereas with ACTB normalization we obtained **p = 0.0011 and *p = 0.0130, respectively. In cscMG-63, for cMyc we obtained ***p = 0.0003 with the new analysis, in place of **p = 0.0019 with the normalization to ACTB.

## Discussion

The evolving concept of CSC has attracted much attention, and the characterization of CSC has led the way to novel prospective for anti-cancer therapies. CSC are a minority subset of tumor-initiating cells involved in various phases of the pro-tumorigenic process, from tumor initiation [30], to chemoresistance [31] and relapse [32]. CSC can be isolated from cell lines and tissue sample with the sphere system method [12], based on the ability of CSC to grow as spherical floating colonies in anchorage-independent conditions. To date, this is considered an invaluable method to obtain CSC-enriched cultures. Recently, we isolated CSC from musculoskeletal sarcomas, both from cell line and fresh biopsy [16,27]. We previously elucidated that CSC and the respective native cells differs not only for the peculiar culture conditions—for the ability to adhere to the substrate or for the addition to the medium of specific nutrients or growth factors—but also and most importantly for morphological and stemness features and lysosomal acidity [16,27], thus probably affecting also energy metabolism.

To study the molecular phenotype of CSC and the underlying cellular mechanism of tumorigenesis, the analysis of gene expression phenotype of native tumor cells and CSC is pivotal. However, despite the recent increasing interest in CSC, an accurate selection of reference genes to a robust normalization of expression data is still missing. The study here presented identified and validated the most stable HKG among 15 candidate reference genes for the normalization of qRT-PCR data. We used a panel of CSC and native cell lines from different human sarcoma histotypes, in particular OS, RS, and ES, including a tumor primary culture derived from a human ES biopsy, and from breast and renal carcinoma. We selected the HKG to be included in this study through a literature survey on the reference genes used for the normalization of qRT-PCR data from normal stem cells [33,34]. Among the normal stem cells, we included mesenchymal stem cells [35,36] that represent the most likely candidate cell of origin for OS and ES [37,38], and tumor cells [39,40].

As a preliminary analysis of the selected candidate reference genes, we used deep sequencing data, and we confirmed that the gene expression profiling of the selected genes were stable, with little or no changes. Then, the expression of selected genes was validated with qRT-PCR, and we found a broad expression pattern. First of all, we analyzed the homogeneity of expression of the selected HKG in CSC and native cells using the paired Wilcoxon signed-rank test. We found significant difference in gene expression for more than a half of the examined HKG. To evaluate the stability of HKG we first analyzed our data by calculating and comparing the coefficient of variation. Afterwards, we validated the results using two well-established algorithms, NormFider and GeNorm. NormFinder determines the stability value using a model-based approach [23]. GeNorm calculates the expression stability of a gene based on the average pairwise variation among all reference genes [22]. The application of 3 different methods of gene stability evaluation could reduce errors associated with a single software, and could avoid the selection of co-regulated transcripts. GeNorm algorithm is highly sensitive to gene co-regulation [22] because, as a pairwise comparison approach is used, co-regulated genes with a similar expression profile obtain best score. The model-based evaluation method of NormFinder avoids misinterpretations that can result from the artificial selection of co-regulated genes, and analyzed the candidate reference genes according to the intra- and inter-group expression variations. Finally, since the use of more than one different HKG is recommended for gene expression normalization [22], we used GeNorm to determine the minimal number of HKG for accurate normalization. The results obtained with the two algorithms, GeNorm and NormFinder, were quite similar. We analyzed the most stable HKG in three groups of data: in CSC, in native cells, and in the CSC and native cells pooled group. By calculating the overall best ranked HKG from the CV, GeNorm and NormFinder analyses we found that PGK1, YWHAZ, and GAPDH were the most stable HKG for the comparison of CSC expression data, whereas the most stable HKG for native cells were PPIA and TBP. When we considered CSC and native cells with all the tumor types pooled together, we identified TBP, PPIA, HMBS and YWHAZ or GAPDH as HKG that should be used for qRT-PCR normalization, also because, by the pairwise variation analysis of the minimum number of genes, we found that at least four genes are required.

The most stable HKG for the CSC and native cells obtained only from sarcoma were GAPDH and YWHAZ, whereas for carcinoma we identified PPIA, HMBS or RPL13a. For comparison of gene expression data from CSC and native cells within the single tumor type, sarcoma or carcinoma, and based on pairwise variation analysis of the minimum number of genes, the use of two HKG was sufficient.

Although with some limits [41], the use as internal standard for Northern blot analysis of the ribosomal subunits 18S rRNA is recommended, since their mRNA variations are weak. Also in our study, 18S RNA resulted as one of the best-ranked housekeeping gene in the pooled group with CSC and native cells of all the cell lines (ranking position 4, 2, 8 in ΔCt, GeNorm, and NormFinder respectively). Therefore, it is likely that for the quantification of mRNA, the use of ribosomal subunits for internal standards is recommended, since it is not highly affected by microenvironmental stimuli and conditions, like in vitro conditions for culturing CSC.

Our evaluation of HKG stability also suggested that ACTB and B2M, two of the most commonly used HKG used in qRT-PCR analyses [20,42], but also RPL13a, ranked among the last position in all the considered conditions, suggesting that these HKG could be unsuitable for the normalization of qRT-PCR data. Adversely, in carcinoma, RPL13a ranked between the most stable HKG in CSC and native cells. Therefore, appropriate HKG should be selected in relation to the tumor investigated. Moreover, the analysis of stemness genes KLF4, c-Myc, Nanog, and OCT3/4 to ACTB rather than to the most stable HKG in CSC and native cells, clearly demonstrated that normalization to the suboptimal HKG can lead to a complete loss of information about the stamness capacity of CSC, or can hide difference in gene expression between CSC and native cells.

Finally, we performed the reference gene stability analysis within spheres and native cells from the different human tumor histotypes considered in this study, and we found considerable variability among the different cell lines in terms of the best HKG (S1 Table). Again, the analysis of the minimum number of gene required for the robust normalization for all the groups considered suggested two HKG (S1 Fig). In particular, based on the average of the HKG ranking obtained from three different methods, we suggested that the optimal normalization factor could be calculated as the geometric mean of GUSB and 18S rRNA for MG-63, PGK1, HMBS or TBP for RD, PPIA and HPRT1 for A-673, YWHAZ and PPIA for ES4540, TBP and YWHAZ for MDA-MB-231, and PPIA and GUSB for ACHN.

Taken together, our results demonstrate that proper reference genes should be appropriately selected under different experimental conditions. We recommend to use at least two of the suggested HKG for normalize qRT-PCR-based gene expression analyses of human CSC and native cells.

## Conclusions

The present study offers substantial information on valid reference genes for the analysis of gene expression variation between CSC and adherent native cells from different types of tumor, providing a valuable platform for transcriptional analyses focused on the pathogenesis of musculoskeletal sarcomas and carcinomas and for identifying specifically anti-tumor therapy targeting CSC.

## Supporting Information

S1 Fig Determination of the minimal number of HKG by pairwise variation in 3 histotypes of sarcoma and 2 of carcinoma. (A) cscMG-63 and MG-63, (B) cscRD and RD, (C) cscA-673 and A-673, (D) cscES4540 and ES4540, (E) cscMDA-MB-231 and MDA-MB-231 and (F) cscACHN and ACHN.
(TIF)

S1 Table NormFinder, geNorm and CV ranking order of candidate reference genes in different histotypes of sarcoma and carcinoma. (DOC)
