# Supplementary figures and images for: Identification and Validation of Housekeeping Genes for Gene Expression Analysis of Cancer Stem Cells
Source: PLoS One. 2016 Feb 19;11(2):e0149481. doi: 10.1371/journal.pone.0149481 (PMC4760967; doi:10.1371/journal.pone.0149481)

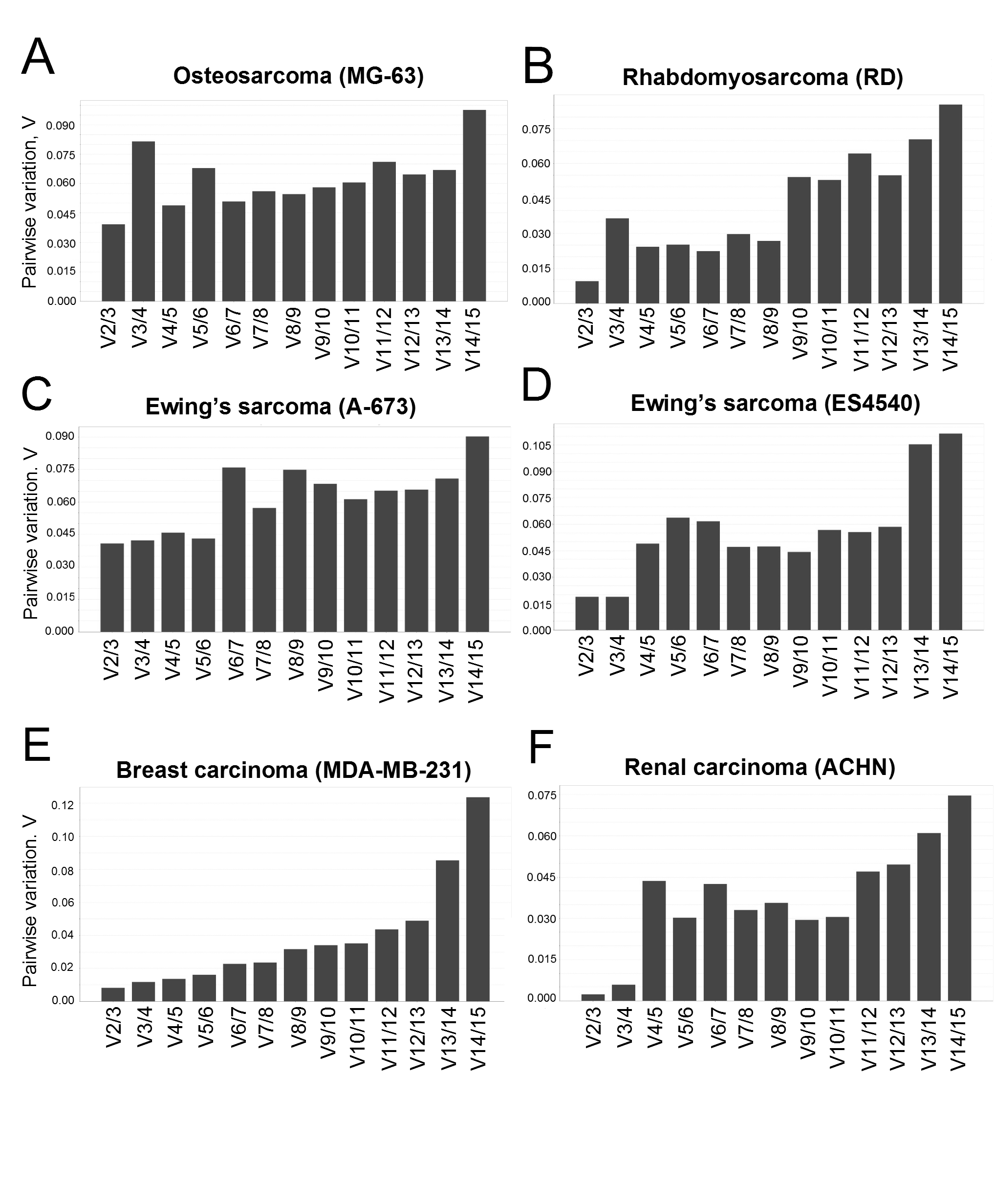

Supplement: S1 Fig — (A) cscMG-63 and MG-63, (B) cscRD and RD, (C) cscA-673 and A-673, (D) cscES4540 and ES4540, (E) cscMDA-MB-231 and MDA-MB-231 and (F) cscACHN and ACHN. (TIF) [file pone.0149481.s001.tif]
