# Supplementary material for: Identification and Validation of Housekeeping Genes for Gene Expression Analysis of Cancer Stem Cells
Source: PLoS One. 2016 Feb 19;11(2):e0149481. doi: 10.1371/journal.pone.0149481 (PMC4760967; doi:10.1371/journal.pone.0149481)
Supplement: S1 Table — (DOC) [file pone.0149481.s002.doc]

**S1 Table. NormFinder, geNorm and CV ranking order of candidate reference genes in different histotypes of sarcoma and carcinoma.**

| **Cell line** | **Gene** | **NormFinder** | | **GeNorm** | | **Coefficient of Variation** | |
| --- | --- | --- | --- | --- | --- | --- | --- |
|  |  | *Stability value* | *Rank* | M value | *Rank* | *CV* | Rank |
| **Osteosarcoma  (MG-63)** | GUSB | 0.125 | 1 | 0.075 | 2 | 0.013 | 1 |
|  | 18S rRNA | 0.236 | 2 | 0.374 | 9 | 0.021 | 3 |
|  | GAPDH | 0.262 | 3 | 0.208 | 5 | 0.036 | 10 |
|  | YWHAZ | 0.265 | 4 | 0.223 | 4 | 0.032 | 8 |
|  | PGK1 | 0.337 | 5 | 0.305 | 7 | 0.033 | 9 |
|  | PPIA | 0.352 | 6 | 0.090 | 3 | 0.037 | 11 |
|  | HPRT1 | 0.399 | 7 | 0.341 | 8 | 0.029 | 7 |
|  | TUBB | 0.416 | 8 | 0.067 | 1 | 0.045 | 13 |
|  | RPL13a | 0.417 | 9 | 0.461 | 11 | 0.024 | 4 |
|  | SDHA | 0.457 | 10 | 0.416 | 10 | 0.025 | 5 |
|  | HMBS | 0.507 | 11 | 0.575 | 13 | 0.017 | 2 |
|  | G6PD | 0.512 | 12 | 0.284 | 6 | 0.038 | 12 |
|  | TBP | 0.519 | 13 | 0.527 | 12 | 0.026 | 6 |
|  | ACTB | 0.557 | 14 | 0.738 | 15 | 0.072 | 15 |
|  | B2M | 0.656 | 15 | 0.627 | 14 | 0.067 | 14 |
| **Rhabdomyosarcoma (RD)** | ACTB | 0.155 | 1 | 0.515 | 14 | 0.040 | 10 |
|  | PPIA | 0.193 | 2 | 0.117 | 6 | 0.038 | 8 |
|  | GAPDH | 0.207 | 3 | 0.128 | 7 | 0.039 | 9 |
|  | PGK1 | 0.236 | 4 | 0.085 | 4 | 0.029 | 7 |
|  | G6PD | 0.238 | 5 | 0.156 | 8 | 0.026 | 4 |
|  | YWHAZ | 0.247 | 6 | 0.099 | 5 | 0.028 | 6 |
|  | HMBS | 0.331 | 7 | 0.024 | 3 | 0.027 | 5 |
|  | B2M | 0.341 | 8 | 0.175 | 9 | 0.046 | 12 |
|  | 18S rRNA | 0.350 | 9 | 0.018 | 1 | 0.051 | 13 |
|  | TBP | 0.351 | 10 | 0.021 | 2 | 0.023 | 3 |
|  | GUSB | 0.372 | 11 | 0.386 | 12 | 0.042 | 11 |
|  | TUBB | 0.384 | 12 | 0.437 | 13 | 0.053 | 14 |
|  | SDHA | 0.410 | 13 | 0.248 | 10 | 0.015 | 1 |
|  | HPRT1 | 0.450 | 14 | 0.309 | 11 | 0.018 | 2 |
|  | RPL13a | 0.602 | 15 | 0.617 | 15 | 0.079 | 15 |
| **Ewing’s sarcoma  (A-673)** | HPRT1 | 0.197 | 1 | 0.296 | 7 | 0.014 | 2 |
|  | PPIA | 0.259 | 2 | 0.097 | 2 | 0.038 | 6 |
|  | G6PD | 0.278 | 3 | 0.174 | 5 | 0.019 | 4 |
|  | YWHAZ | 0.315 | 4 | 0.202 | 6 | 0.031 | 5 |
|  | TBP | 0.326 | 5 | 0.466 | 10 | 0.014 | 1 |
|  | PGK1 | 0.353 | 6 | 0.105 | 3 | 0.039 | 8 |
|  | SDHA | 0.361 | 7 | 0.079 | 1 | 0.040 | 10 |
|  | GAPDH | 0.366 | 8 | 0.137 | 4 | 0.049 | 12 |
|  | ACTB | 0.374 | 9 | 0.412 | 9 | 0.049 | 13 |
|  | 18S rRNA | 0.396 | 10 | 0.504 | 11 | 0.068 | 15 |
|  | HMBS | 0.480 | 11 | 0.597 | 13 | 0.039 | 9 |
|  | GUSB | 0.513 | 12 | 0.550 | 12 | 0.017 | 3 |
|  | RPL13a | 0.549 | 13 | 0.337 | 8 | 0.044 | 11 |
|  | TUBB | 0.596 | 14 | 0.654 | 14 | 0.053 | 14 |
|  | B2M | 0.769 | 15 | 0.747 | 15 | 0.039 | 7 |
| **Primary Ewing’s sarcoma (ES4540)** | PPIA | 0.146 | 1 | 0.135 | 5 | 0.038 | 5 |
|  | GAPDH | 0.213 | 2 | 0.049 | 3 | 0.043 | 7 |
|  | YWHAZ | 0.244 | 3 | 0.037 | 1 | 0.036 | 4 |
|  | B2M | 0.244 | 4 | 0.279 | 7 | 0.052 | 13 |
|  | TBP | 0.275 | 5 | 0.303 | 8 | 0.027 | 2 |
|  | HMBS | 0.276 | 6 | 0.331 | 9 | 0.032 | 3 |
|  | 18S rRNA | 0.282 | 7 | 0.218 | 6 | 0.110 | 15 |
|  | SDHA | 0.291 | 8 | 0.353 | 10 | 0.044 | 9 |
|  | HPRT1 | 0.353 | 9 | 0.062 | 4 | 0.023 | 1 |
|  | TUBB | 0.353 | 10 | 0.045 | 2 | 0.044 | 10 |
|  | PGK1 | 0.467 | 11 | 0.402 | 11 | 0.040 | 6 |
|  | ACTB | 0.478 | 12 | 0.635 | 14 | 0.044 | 8 |
|  | GUSB | 0.485 | 13 | 0.495 | 13 | 0.051 | 12 |
|  | RPL13a | 0.501 | 14 | 0.447 | 12 | 0.045 | 11 |
|  | G6PD | 0.936 | 15 | 0.773 | 15 | 0.056 | 14 |
| **Breast carcinoma (MDA-MB-231)** | 18S rRNA | 0.018 | 1 | 0.014 | 1 | 0.165 | 15 |
|  | TBP | 0.043 | 2 | 0.115 | 8 | 0.064 | 2 |
|  | HMBS | 0.051 | 3 | 0.226 | 11 | 0.082 | 7 |
|  | PPIA | 0.061 | 4 | 0.047 | 5 | 0.082 | 8 |
|  | RPL13a | 0.069 | 5 | 0.019 | 3 | 0.088 | 10 |
|  | G6PD | 0.077 | 6 | 0.191 | 10 | 0.080 | 5 |
|  | YWHAZ | 0.116 | 7 | 0.016 | 2 | 0.081 | 6 |
|  | HPRT1 | 0.118 | 8 | 0.276 | 12 | 0.087 | 9 |
|  | GAPDH | 0.120 | 9 | 0.033 | 4 | 0.091 | 12 |
|  | ACTB | 0.125 | 10 | 0.331 | 13 | 0.115 | 13 |
|  | GUSB | 0.153 | 11 | 0.091 | 7 | 0.075 | 4 |
|  | B2M | 0.157 | 12 | 0.642 | 15 | 0.034 | 1 |
|  | SDHA | 0.181 | 13 | 0.064 | 6 | 0.073 | 3 |
|  | TUBB | 0.194 | 14 | 0.455 | 14 | 0.141 | 14 |
|  | PGK1 | 0.203 | 15 | 0.153 | 9 | 0.089 | 11 |
| **Renal carcinoma (ACHN)** | PPIA | 0.014 | 1 | 0.174 | 7 | 0.023 | 3 |
|  | GAPDH | 0.034 | 2 | 0.096 | 5 | 0.038 | 11 |
|  | ACTB | 0.047 | 3 | 0.121 | 6 | 0.037 | 9 |
|  | PGK1 | 0.049 | 4 | 0.197 | 8 | 0.031 | 8 |
|  | GUSB | 0.056 | 5 | 0.005 | 1 | 0.026 | 5 |
|  | HPRT1 | 0.067 | 6 | 0.015 | 4 | 0.027 | 6 |
|  | HMBS | 0.071 | 7 | 0.006 | 2 | 0.022 | 2 |
|  | 18S rRNA | 0.086 | 8 | 0.238 | 10 | 0.064 | 15 |
|  | G6PD | 0.087 | 9 | 0.224 | 9 | 0.008 | 1 |
|  | TUBB | 0.104 | 10 | 0.430 | 14 | 0.056 | 14 |
|  | RPL13a | 0.106 | 11 | 0.006 | 3 | 0.041 | 13 |
|  | TBP | 0.114 | 12 | 0.307 | 12 | 0.025 | 4 |
|  | B2M | 0.120 | 13 | 0.359 | 13 | 0.029 | 7 |
|  | YWHAZ | 0.191 | 14 | 0.256 | 11 | 0.037 | 10 |
|  | SDHA | 0.243 | 15 | 0.522 | 15 | 0.039 | 12 |
